# Unanswered questions about the Middle East respiratory syndrome coronavirus (MERS-CoV)

**DOI:** 10.1186/1756-0500-7-358

**Published:** 2014-06-11

**Authors:** Lauren M Gardner, C Raina MacIntyre

**Affiliations:** 1 CE 112 School of Civil and Environmental Engineering, The University of New South Wales, Sydney, NSW 2052, Australia; 2 NICTA, Sydney, NSW 2052, Australia; 3 School of Public Health and Community Medicine, Faculty of Medicine, University of New South Wales, Sydney, NSW 2052, Australia

**Keywords:** MERS-CoV, Emerging infectious disease, Epidemiology

## Abstract

**Background:**

The Middle East respiratory syndrome coronavirus (MERS-CoV) represents a current threat to the Arabian Peninsula, and potential pandemic disease. As of June 3, 2014, MERS CoV has reportedly infected 688 people and killed 282. We briefly summarize the state of the outbreak, and highlight unanswered questions and various explanations for the observed epidemiology.

**Findings:**

The continuing but infrequent cases of MERS-CoV reported over the past two years have been puzzling and difficult to explain. The epidemiology of MERS-CoV, with many sporadic cases and a few hospital outbreaks, yet no sustained epidemic, suggests a low reproductive number. Furthermore, a clear source of infection to humans remains unknown. Also puzzling is the fact that MERS-CoV has been present in Saudi Arabia over several mass gatherings, including the 2012 and 2013 Hajj and Umrah pilgrimages, which predispose to epidemics, without an epidemic arising.

**Conclusions:**

The observed epidemiology of MERS-CoV is quite distinct and does not clearly fit either a sporadic or epidemic pattern. Possible explanations of the unusual features of the epidemiology of MERS-CoV include sporadic ongoing infections from a non-human source; human to human transmission with a large proportion of undetected cases; or a combination of both. The virus has been identified in camels; however the mode of transmission of the virus to humans remains unknown, and many cases have no history of animal contact. In order to gain a better understanding of the epidemiology of MERS CoV, further investigation is warranted.

## Findings

Emerging infectious diseases are a constant threat to human health. Of specific concern is the uncertainty associated with their early stages and potentially high impact outcome. In April 2012, the Middle East respiratory syndrome coronavirus (MERS-CoV) emerged [1] and spread from the Arabian Peninsula to various countries in Europe, North Africa, Southeast Asia, the United States and Middle East. MERS-CoV is characterized by a severe respiratory illness and high case-fatality rate [2]. A decade prior, a pandemic of SARS, a related coronavirus, caused 8,273 cases and 775 deaths in only 8 months [3]. In contrast, MERS-CoV is still ongoing after two years, and as of June 2014 has apparently infected only 688 people and killed 282, with no clear source of infection to humans and an epidemiologic pattern that is more sporadic than the classic epidemic pattern of SARS [2]. Of further concern is the significant increase in reported cases since March 2014; in April 2014 alone, the number of reported cases exceeded the total number of cases that had been reported in the two years prior [2].

The first outbreak of MERS-CoV occurred in a hospital in Jordan in April 2012, with subsequent cases and clusters occurring throughout the Arabian Peninsula. Phylogenetic analysis of the virus has revealed that MERS-CoV has been in circulation since at least 2003 [4], much longer than previously estimated based on the most common ancestor for the MERS-CoV strains found in humans [5], and well before the first confirmed case in Jordon. Travel from the infected regions has resulted in additional cases in over a dozen countries, with limited local transmission occurring in the UK, France, Tunisia, and Iran. Furthermore, five of the highest travelled airports in the world are located in countries where MERS-CoV has been transmitted [6], creating the possibility of a pandemic [7].

Hospital clusters represent the majority of MERS-CoV confirmed cases, and are the primary location where human-to-human transmission of MERS-CoV has been confirmed [8-10]; although limited spread among family members has also been confirmed [11]. SARS was also predominantly a nosocomial infection [12], but the epidemiology of MERS-CoV is less clear. The risk factors associated with MERS-CoV include male gender, underlying disease, immunosuppression and hospitalization. These patterns contrast the epidemiology of SARS, where there was a small female excess, less evidence of co-morbidities, and a younger median age. The male predominance of MERS-CoV may reflect higher likelihood of exposure to infectious agents for males than females in Middle Eastern cultures. In addition, most males who tested positive for the virus and died also had underlying medical conditions, which could explain the severity of cases in older males. Asymptomatic, child and female cases have been increasingly recently reported [9], however a large number of undetected asymptomatic or mild cases have not been found during contact tracing among healthcare workers or close contacts of MERS-CoV patients at the German [13], UK [14] and KSA hospitals [1], nor in a serologic survey conducted on blood donors and abattoir workers in the infected region in 2012 [15].

In contrast to SARS which was rapidly identified as zoonotic in origin [3], there has been no clear animal source of infection, nor consistent history of animal contact in a majority of the reported cases. Phylogenetic analysis has identified a close relationship between MERS-CoV in humans and various bat species [16-19], however the exact virus has not been confirmed in any bat. In contrast, MERS-CoV and MERS-CoV-like antibodies have been identified in dromedary camels [4,20-31], suggesting MERS-CoV is widespread, and previously infected various camel populations (including countries where human cases have yet to be reported). A recent study by Memish et al. [32] analyzed the virus in an infected camel and infected care taker of the camel, and the findings suggested cross-species transmission, though it is unknown if the camel infected the human, or the other way around. Infected camels may therefore represent a direct source to humans, or the virus may have crossed from camels to alternative zoonotic hosts or environmental sources responsible for the recent transmission to humans. Additional evidence supporting camels as a host animal was provided by a study which sequenced complete MERS-CoV isolates from five camels in Saudi Arabia, which were shown to be identical to published sequences of human isolates [31]. The same study found that viral particles from individual camels contained more genetic variation than MERS-CoV isolates from humans; therefore only certain genotypes may be able to infect humans, offering a possible explanation to why human cases are less common [31].

**Figure 1:**
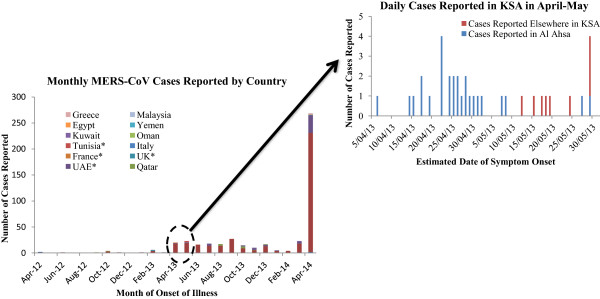
**Case reports of MERS-CoV.** In the main graph the total monthly cases reported by Country are shown, where *indicates local transmission occurred. The inset illustrates the number of daily cases reported in KSA in April and May in Al Ahsa. Data provided by WHO [2].

With MERS CoV there was no epidemic peak in the first 12 months as there was with SARS [12]. Instead, there were infrequent cases over a longer period than would be expected for a disease that is not highly infectious (see Figure 1). This pattern is suggestive of a sporadic rather than epidemic source of ongoing infection in humans. Estimates of the reproductive number, R0, are low, and range from 0.42 < R0 < 0.92 [33], and 0.8 < R0 < 1.5 [34], suggesting the infection does not have epidemic potential and cannot be easily sustained in the human population by human-to-human transmission alone [9,35,36]. A low reproductive number is supported by the epidemiologic pattern, however, the surge of cases in April 2014, inclusion of asymptomatic and mild infections and the increasing size and transmission generations of recent clusters would under-estimate this calculation.

At odds with these estimates of R0, in April and May 2013, a peak of infections occurred in Al Ahsa (see Figure 1). This outbreak resulted in 26 cases, and shows a classic epidemic curve characteristic of an infection with a R0 > 1, which on the surface can be interpreted as a single strain human-to-human, rapid-onset, short time- frame nosocomial outbreak [37]. Yet phylogenetic analysis of the patient samples showed multiple different strains, and at least three transmission events which could not be explained by human-to-human transmission within the hospital [38]. These data suggests the outbreak was a result of multiple independent transmission events, combined with some human-to-human transmission within the hospital [38]. Multiple sources of transmission were again identified in a study of the Hafr Al-Batin outbreak conducted by Memish et al. [39], with camels suggested as the most likely source.

In yet another paradox, given apparent human-to-human transmission in Al Ahsa and Hafr Al-Batin, among others, it is puzzling that MERS-CoV was present in Saudi Arabia around the period of significant mass gatherings such as the Umrah and Hajj pilgrimages in 2012 and 2013, yet no cases were reported in returning pilgrims either year [2]. The Hajj is a significant mass gathering, with over four million pilgrims congregating in Mecca, and is associated with significant epidemics of infectious diseases [40]. Active surveillance of symptomatic pilgrims in 2012 and 2013 failed to detect MERS-CoV infections [41-44], a serologic survey conducted on blood donors and abattoir workers in the infected region in 2012 did not detect any antibodies [15]. Additionally, no cases were reported by pilgrims who travelled to Saudi Arabia to perform Umrah during July and August, 2013.The lack of an epidemic sparked by the close contact conditions of four mass gatherings in Saudi Arabia during 2012 and 2013 supports the low estimates of R0.

The persistence of MERS-CoV for more than 3 times the duration than SARS, which had a higher R0, highlights the question of why the infection is still ongoing in humans, and what is the source of ongoing transmission to humans? The possible sources of ongoing infection include a non-human source which is infecting humans (such as an animal reservoir or an environmental source), or human-to-human transmission. Human to human transmission is apparent in some clusters of individuals in close contact and in the hospital setting, but does not appear frequent, supporting a low infectivity of the virus. While it is possible MERS-CoV has been circulating solely from sustained human to human transmission since it was initially reported in mid 2012, it is unlikely given the low number of reported cases. It is possible that cases have been either asymptomatic or escaped detection, as cases with mild or no symptoms do appear to occur. Undetected mild cases have been suggested as a factor in the apparent decrease in the case fatality rate over time in a model estimating a total of 940 (95% CI 290–2200) symptomatic cases, with 62% of these undetected [45]. However, efforts to identify a large pool of undetected cases have failed to identify asymptomtic or mild cases [1,13,14], and similarly, a serological study showed no evidence that MERS-CoV circulated widely in the study region in fall 2012 [15]. In other words, the model [45] can only explain the observed epidemiology if a large proportion of undetected cases have occurred, yet there is no evidence to support this. Based on the available evidence, multiple introductions from zoonotic sources to humans, resulting in limited clusters of cases is the most likely explanation of how MERS CoV has persisted in human populations for a prolonged period despite a low R0, although it is unknown how many jumps to humans have occurred, or what the mode of transmission is between the animals and humans [34].

Clearly, there are still unanswered questions and unusual features of the epidemiology of MERS-CoV which warrant ongoing investigation. Given the remaining uncertainties surrounding MERS CoV, and the lack of an available vaccine, a strategic approach is required to control future spread. Firstly, zoonotic sources of infection should continue to be investigated, with a focused interest in identifying the possible zoonotic hosts or environmental sources which may act as modes of transmission between camels and humans. A protocol has been adopted by WHO with the primary purpose of determining the non-human source of infection and corresponding route and mode of transmission to humans. Second, genetic testing over time should be conducted to determine if the virus is evolving, in which case the epidemiology of MERS-CoV could significantly alter. The outbreak in Al Ahsa may reflect such a change, as could the spike of cases observed in April, 2014. The required serological tools for the detection of specific MERS-CoV antibodies (e.g. ELISA) have been developed and validated, and are currently being administered [32]. Further serological surveys, contact tracing and other surveillance in affected areas are needed to quantify asymptomatic or mild infection and to identify exposures to non-human sources of infection. Third, the possibility of a large number of cases having escaped detection, resulting in a falsely skewed epidemiology must be considered. Based on the viral load of MERS-CoV, the shedding patterns of MERS-CoV are different than those of SARS, possibly requiring alternative diagnostic approached [10]. Further inquiry should also address the difference in symptoms between cases resulting from person-to-person transmission compared with transmission from unknown sources, presumably non-human, which may be more virulent; however a larger sample of case data is required to confirm this phenomenon. Lastly, open international collaboration and public access to laboratory findings from surveys such as that by ECDC-WHO can reveal the true geographical extent and source of these infections.

## Competing interests

The authors declare there is no conflict of interest.

## Authors’ contributions

Both authors contributed equally to the writing of this manuscript. Both authors read and approved the final manuscript.
